# Therapeutic Potential of Circular RNAs in Osteosarcoma

**DOI:** 10.3389/fonc.2020.00370

**Published:** 2020-04-15

**Authors:** Ben Wan, Hao Hu, Renxian Wang, Weifeng Liu, Dafu Chen

**Affiliations:** ^1^Laboratory of Bone Tissue Engineering, Beijing Laboratory of Biomedical Materials, Beijing Research Institute of Traumatology and Orthopaedics, Beijing Jishuitan Hospital, Beijing, China; ^2^Guangdong Provincial Key Laboratory of Orthopaedics and Traumatology, Guangzhou, China; ^3^Department of Spinal Surgery, The First Affiliated Hospital of Sun Yat-sen University, Guangzhou, China; ^4^Department of Orthopaedic Oncology Surgery, Beijing Jishuitan Hospital, Peking University, Beijing, China

**Keywords:** natural circular RNA, artificial circular RNA, osteosarcoma, RNA-based therapy, cancer therapeutic strategy

## Abstract

Osteosarcoma is the most common malignant bone tumor in children and adolescents. Multiagent chemotherapy, together with surgical removal of all detectable lesions, has improved the long-term survival rate to 65–70% in patients with localized osteosarcoma and to 25–30% in patients with metastatic osteosarcoma since the 1970s. However, the conventional strategy has not improved in recent decades. With accumulating knowledge of the natural circular RNA (circRNA) pathogenesis of osteosarcoma, the diagnostic and therapeutic potential of some circRNAs has been explored. Meanwhile, artificial circular RNAs have been designed as onco-microRNA inhibitors to exert antitumor functions. Therefore, natural and artificial circular RNAs, like other RNA counterparts, are attractive new classes of therapeutic molecules for the treatment of osteosarcoma. This review summarizes the latest progress in the relationship between circRNAs and the malignant phenotype of osteosarcoma and sheds light on the therapeutic potential of the two types of circular RNA in the clinic.

## Introduction

Osteosarcoma (OS) is a rare disease (incidence: 0.3 per 100,000 people per year) but has a high prevalence in children and adolescents. Most tumors arise in an extremity, while the proportion of axial tumor sites increases with age (1). It is a neoplasm that derives from primitive bone-forming mesenchymal cells that have histological evidence of osteoid production (2). The majority of primary OSs are high-grade bone tumors that account for 80–90% of all OSs (3). Due to the diverse molecular pathogeneses, lack of useful biomarkers, high local aggressiveness, and rapid metastasizing potential, traditional treatments have encountered serious setbacks (4). Although intensive adjuvant chemotherapy combined with surgery has achieved a 5-year survival rate of 60–70% in extremity-localized OS, the overall 5-year survival rate for patients with metastatic or relapsed OS has been around 20% for the past 30 years, and relapse rates have remained high at ~35% (5). Multidrug resistance and metastasis have long been two major obstacles in the treatment of OS and are also important causes of poor prognosis.

Initially thought to be the outcome of transcriptional noise, many non-coding RNAs (ncRNAs) have been found to participate in the pathogenesis of OS. Unlike microRNAs (miRNAs) and long non-coding RNAs (lncRNAs) that are already established research subjects, natural circular RNAs (circRNAs) are gaining increasing attention in the cancer research field. In general, the universal downregulation of circRNAs has been discovered in various tumors, which means that circRNAs may be diagnostic or therapeutic targets across cancer types (6, 7). Several reports have found that circRNAs are involved in the progression, metastasis, and multidrug resistance of OS (8, 9). As for the regulatory mechanism, it is widely accepted that circRNAs can act as competing endogenous RNAs (ceRNAs) to inhibit the function of certain endogenous miRNAs. Indeed, evidence has revealed that a ceRNA interacting network plays a vital role in regulating the pathogenic process of OS (10, 11). Additionally, newly developed artificial circular RNA molecules have been designed as exogenous miRNA inhibitors that effectively bind and block mature miRNAs, showing promise for the molecular therapy of cancer (12). While natural circRNAs found in the pathogenesis of OS may provide novel therapeutic targets, artificial circular RNAs designed for specific onco-microRNAs may also provide novel RNA-based therapy. Here, we review the role of circRNAs in the pathogenesis of OS with emphasis on the therapeutic potential of both natural and artificial circRNAs.

## General Features of circRNAs

There are different types of circRNAs that are generated by distinct mechanisms, including circular RNA of the genomes of viroids, intermediates in rRNA processing or permuted tRNAs, and products of back-splicing of pre-mRNAs in eukaryotes (13). The most currently well-studied circRNAs generate their cyclic conformation from linear pre-mRNAs that back-splice or form exon lariats in which the 5′ splice site (splice donor) is joined to an upstream 3′ splice site (splice acceptor), resulting in a circular RNA with ends that are covalently ligated by a 3′-5′ phosphodiester bond (14).

CircRNAs have a unique circular structure that is resistant to degradation by most RNA decay machinery. Hansen et al. reported that the degradation of the circular CDR1 antisense transcript relies on the miR-671-mediated, Ago2-dependent cleavage pathway (15, 16). However, whether other circRNAs undergo a similar cleavage mechanism remains unknown. As opposed to their associated linear transcripts, circRNAs are characterized by a continuous covalently closed loop without 5′-3′ polarity or a 3′-poly A tail, giving them stronger resistance to RNase R and longer half-lives (17). It has been revealed that the median half-lives of circRNAs of mammary cells (18.8–23.7 h) are at least 2.5 times longer than their linear counterparts transcribed from the same host gene (18). CircRNAs exist stably in serum exosomes and human cell-free saliva, indicating their potential to be reliable biomarkers (19, 20). Such inherent stability makes this class of RNA a strong candidate to maintain homeostasis in the face of environmental challenge.

The potential mechanisms of circRNAs as miRNA inhibitors have been extensively explored. Pandolfi et al. proposed a ceRNA hypothesis in which different RNA transcripts interact with each other by competing for miRNAs through miRNA recognition elements (MREs) (21). Endogenous circRNAs could regulate gene expression by sequestering miRNAs that mediate negative regulation of their target genes (16, 22, 23). Rajewsky's and Kjems's labs have provided evidence supporting this idea, focusing on a natural antisense transcript (CDR1as) to cerebellar degeneration related protein 1 (CDR1) (16, 24). In contrast to other circular natural antisense transcriptional products, CDR1as contains about 70 MREs for miR-7 and 1 for miR-671. CDR1as functions as an miRNA antagonist, similar to knocking-down miR-7, impairing midbrain development in a zebrafish model (24). Knockdown of CDR1as in OS, hepatocellular carcinoma, and colorectal cancer cell lines leads to de-repressed miR-7 levels, downregulating miR-7-target genes and impairing cell vitality (25–27). Piwecka et al. reported the first CDR1as knockout mouse model and surprisingly observed that miR-7 was markedly downregulated, while its target genes were specifically upregulated in the brain (28). Bezzi et al. found that the prolonged absence of “sponge” circRNAs leads to destabilization of their binding miRNAs and de-repression of their targets (22). These studies imply that circRNAs may also serve as competing endogenous RNAs to store and transport miRNAs like a storage pool, indicating that they may serve a regulatory function in OS.

## CircRNAs Involved in Malignant Phenotypes of OS

Although the fundamental molecular mechanisms underlying tumorigenesis, drug resistance, and metastasis of OS remain obscure, some studies have shown a clear relationship between certain circRNAs and malignant phenotypes (Table 1), which may provide new therapeutic targets for OS patients.

**Table 1:** Roles of circRNAs in the development of osteosarcoma.

**Phenotypes**	**CircRNA**	**Pathway/target**	**References**
**Upregulated circRNAs**			
Tumorigenicity	Hsa_circ_0001564	Hsa_circ_0001564/miR-29c-3p	(29)
	Hsa_circ_0009910	Has_circ_0009910/miR-449a/IL6R axis.	(30)
	CDR1as	CDR1as/miR-7 signals	(25)
Drug-resistance	circPVT1	Increasing the expression of ABCB1	(31)
	Has_circ_001569	Activating Wnt/β catenin signaling	(32)
	Hsa_circ_0004674	Has circ 0004674/miR-490-3p/ABCC2 Has circ 0004674/miR-1254/EGFR	(33)
	Hsa_circ_0001258	Hsa_circ_0001258/hsa-miR-744-3p/GSTM2	(10)
Metastasis	circTADA2A	circTADA2A/miR-203a-3p/CREB3	(34)
	Has_circ_0016347	Has_circ_0016347/miR-214/caspase-1	(35)
	circNASP	circNASP/miR-1253/FOXF1	(36)
**Downregulated circRNAs**			
Tumorigenicity	circHIPK3	–	(37)
	Hsa_circ_0002052	Hsa_circ_0002052/miR-1205/APC2	(38)

### CircRNA-Mediated Chemoresistance of OS

A major obstacle for OS therapy is resistance to chemotherapy, which occurs in 35–45% of patients (39). The prognosis of patients with metastatic disease remains poor, with the 5-year survival rate less than 20% despite aggressive therapy. Therefore, understanding the mechanisms of chemoresistance is conducive to developing novel anti-OS strategies. CircRNA dysregulation related to chemotherapeutic resistance in OS has been reported with the help of high-throughput RNA sequencing, and the underlying mechanisms have been illustrated.

Zhang et al. reported that hsa_circ_001569 is significantly upregulated in OS tissues and facilitates enhanced resistance to cisplatin by activating the Wnt/β-catenin pathway (32). Zhu et al. found that knocking-down circPVT1 partly reversed the chemoresistance to doxorubicin and cisplatin by reducing the expression of ABCB1, a typical multidrug resistance-related gene (31). Zhu et al. further revealed the possible mechanisms of chemoresistance in OS in novel ways. They compared three paired multidrug-resistant and -sensitive OS cell lines by next-generation sequencing and found that hsa_circ_0004674 is distinctly upregulated in drug-resistant OS cell lines and in chemoresistant OS tissue. Furthermore, they speculated that hsa_circ_0004674 mediates chemoresistance by regulating the circRNA/miR-490-3p/ABCC2 or circRNA/miR-1254/EGFR axis by searching bioinformatics databases (Target Scan and miRanda) (33). To further elucidate the circRNA drug-resistance mechanism, Zhu et al. developed whole-transcriptome sequencing in multiple drug-resistant OS cell lines to identify differentially expressed ncRNAs and mRNAs, validating two ceRNA regulatory pathways, namely lncRNAMEG3/hsa-miR-200b-3p/AKT2 and hsa_circ_0001258/hsa-miR-744-3p/GSTM2 regulatory axes. In conclusion, circRNAs participate in the development of chemoresistance in OS by modulating signaling pathways or by their involvement in ceRNA regulatory networks, and therefore could potentially serve as novel therapeutic targets.

### CircRNA-Mediated Metastasis of OS

OS is prone to metastases, which mostly arise in lungs (85–90%) but also develop in bones (8–10%) and rarely in lymph nodes (3). Approximately 20–25% of patients are diagnosed with lung metastasis before the initial treatment, and about 80% of patients progress with metastases following surgical resection (40, 41). Current treatment for patients with pulmonary metastasis is radical metastasectomy combined with chemotherapy. However, it fails to prolong the long-term survival rate. Once the disease has advanced, prognosis is poor with a survival rate of less than 20%. Recent work has shown that circRNAs crosstalk with regulatory miRNAs in OS metastasis. Wu et al. systemically elucidated the role of circTADA2A in the progression and metastasis of OS. They found that circTADA2A acted as an oncogene by sponging miR-203a-3p and upregulating the expression of CREB3. CREB3, a driver gene, enhances the expression of mmp-9 and Bcl-2, resulting in progression and metastasis in OS (34). In a similar pro-metastatic mechanism, Huang et al. reported that the expression level of circNASP is positively correlated with metastasis in OS patients and modulates malignant behaviors via the miR-1253/FOXF1 axis (36). As illustrated in Table 1, the aberrant expression of circRNAs is closely related to metastatic tendencies.

## Clinical Implications of circRNA

OS is an aggressive malignancy with poor clinical outcomes. Thus, it is urgent to identify reliable and non-invasive biomarkers to detect the disease at a very early stage. Endogenous circRNAs are ideal biomarkers for the diagnosis and prognosis of OS due to their stability in serum exosomes.

MiRNAs are endogenous noncoding RNAs closely related to the development of OS, and their aberrant expression results in carcinogenesis and cancer progression (42–44). CircRNAs have been proven to be effective inhibitors of miRNAs and can rescue their abnormal expression, ultimately stopping the cancer process.

### Endogenous circRNAs as Potential Biomarkers

Histological evaluation is the gold standard for diagnosing OS, but the method is invasive and sometimes too late (45). CircRNAs are highly dysregulated in OS and can be detected in tissues and even in plasma samples, which make them ideal candidates for biomarkers. For instance, Zhu et al. confirmed that the upregulation of serum circPVT1 can distinguish OS patients from healthy individuals, finding that circPVT1 is more reliable than alkaline phosphatase for diagnosis (31). In another study, Zhang et al. revealed that circUBAP2 expression is significantly positively correlated with the tumor stages of OS, and Kaplan-Meier survival analysis showed that increased circUBAP2 correlates with reduced survival and poor prognosis (46). Huang et al. conducted a meta-analysis of abnormal circRNAs in OS patients (47). In terms of prognosis, both oncogenic and tumor-suppressor circRNAs had effects on overall survival. In the diagnosis of OS, they calculated an area under the curve of 0.85, with 80% sensitivity, and 77% specificity, using circRNAs.

### Endogenous circRNAs as Targets for OS Therapy

CircRNAs, as ceRNAs, are natural miRNA inhibitors that bind to a limited miRNA pool to relieve the repression of target RNAs. Endogenous circRNAs function as either tumor activators or tumor suppressors in OS (25, 38). These opposing effects give circRNAs great potential for therapeutic strategies. Most reported circRNAs are carcinogenic, playing essential roles in chemoresistance and metastasis of OS (Table 1). These can be targeted and knocked down to impair the development of OS and have long been used for rescue experiments. RNA interference (RNAi) is the most common approach via targeting back-splicing junction sites in order to correct the expression of carcinogenic circRNAs (48). Some circRNAs are downregulated in OS, and overexpressing them by transfection significantly suppresses OS progression. Hsa_circ_0002052 and circHIPK3 are significantly deregulated in OS tissues and cell lines and act as tumor suppressor genes that may serve as therapeutic molecules for OS (37, 38). Taken together, circRNAs can either serve as oncogenic stimuli or tumor suppressors in OS and are therefore potential therapeutic targets for OS intervention.

### Artificial circRNAs as Novel miRNA Inhibitors

With the increasing demand for regulating miRNA activity for cancer therapies, a newly designed artificial miRNA sponge has been developed. Inspired by natural circRNAs reported as endogenous miRNA sponges, a practical artificial circRNA sponge has been synthesized using simple enzymatic ligation steps (12). The artificial circRNA has five bulged miR-21 binding sites that can sponge multiple miR-21 molecules, and better restoration of luciferase activity has been observed as compared with a conventional linear miR-21 inhibitor. Its linear counterparts degrade by 92% in a condition of 4% FBS after 30 min, while the synthetic circRNA only degrades by 9% (12). The sponge not only avoids degradation by exonucleases but also upregulates the expression of the tumor suppressor gene DAXX targeted by miR-21, thus significantly inhibiting the proliferation of gastric cancer cells (12). Liu et al. constructed circRNA sponges for miR-21 and miR-221 using a circular sponge-producing vector, and these were found to be more effective in repressing miRNA targets and anticancer activities compared to typical linear miRNA sponges in malignant melanoma cell lines (49). Shu et al. developed an intracellular vector to express circular miRNA inhibitors, anti-miR223 and anti-miR21, which inhibit the function of carcinogenic miRNAs more effectively than their linear counterparts (50). These custom circular sponges provide a new means of inhibiting the function of targeted miRNAs *in vitro*.

### The Advantages of circRNAs

Their circular conformation gives circRNA molecules some advantages over other miRNA inhibitors. (1) CircRNAs are stable in the cytoplasm and resist degradation by RNA enzymes and miRNAs (17, 24, 48). Moreover, circRNAs function rapidly and efficiently by affecting hundreds of transcription products by regulating their matching miRNAs (51). (2) Compared with manmade anti-miRNA oligonucleotides (AMO), artificial circRNA sponges offer greater clinical value. AMOs are 17–22 nt single-stranded antisense oligonucleotides that act against natural miRNAs and are chemically modified and optimized for *in vivo* delivery (52). However, they fail to maintain a relatively high concentration for satisfactory suppression in the absence of continuous supply. In contrast, miRNA sponges can exert prolonged or permanent suppression against targeted individual miRNAs or against an entire miRNA family by recognizing shared MREs (53–55). In 2018, Liu et al. and Josta et al. provided a proof-of-principle that artificial circRNAs can sponge targeted miRNAs and reduce a series of post-transcriptional products more efficiently compared with conventional AMOs (12, 56, 57).

## Perspectives and Conclusion

Since the first RNAi-based oligonucleotide was approved by the United States Food and Drug Administration in 2018, many pharmaceutical companies have raced to develop RNAi-based drugs (58). RNAi-based drugs directly bind to their targets with perfect complementarity and mediate the cleavage of specific transcripts, while miRNA-based therapies aim to reestablish normal expression and function of key miRNAs (59). Even though no miRNA-based therapeutics have been approved for clinical use, clinical trials support the validity of miRNA inhibition in clinical applications and might inspire future attempts to develop antagomiRs or miRNA mimics for use in cancer therapy (60, 61).

In terms of miRNA-based agents in OS, many miRNAs with great therapeutic properties have been developed in preclinical research. However, none of them have yet reached the clinical evaluation stage. For instance, miR-21 is a typical oncogenic miRNA that is overexpressed in many types of tumors and is involved in all tumor stages ranging from initiation to metastasis (62, 63), including in OS. Chen et al. reported that miR-21 promotes proliferation and invasion by activating the PTEN/Akt pathway (64). Wu et al. revealed that miR-21 negatively regulates the tumor suppressor gene RECK, leading to cell invasion and migration (65). Yang et al. showed that miR-21 regulates sensitivity to cisplatin (66), and Xu et al. discovered that miR-21 inhibits the apoptosis of OS by downregulating caspase 8 (67). Ren et al. reported that high miR-21 expression in tumor tissues is associated with poor prognosis and shorter survival (68). These findings raise concerns about the use of anti-miR-21 as a therapeutic agent in OS.

**Figure 1:**
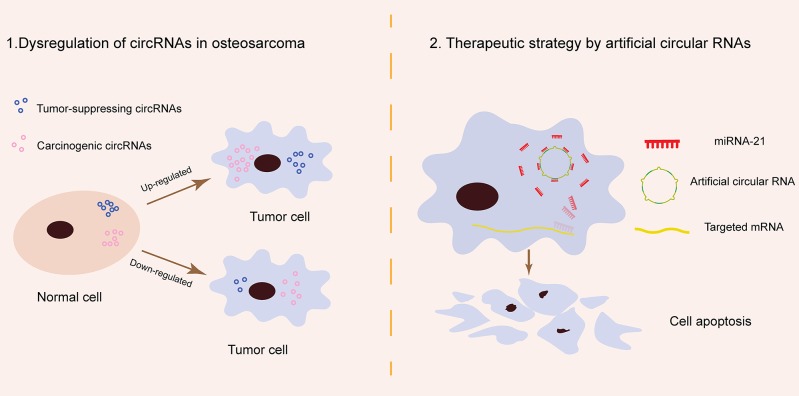
Schematic of circRNA pathogenesis in osteosarcoma and their potential role in osteosarcoma therapy. (1) Tumor-suppressing and carcinogenic circRNAs are naturally present in normal cells and remain in relatively stable amounts. In osteosarcoma, tumor-suppressing circRNAs are downregulated while carcinogenic circRNAs are upregulated. The dysregulation of specific natural circRNAs contributes to osteosarcoma carcinogenesis. (2) miR-21 is a typical oncogenic miRNA in osteosarcoma. Artificial circular RNAs produced by chemical synthesis may block osteosarcoma progress by sponging miRNA-21 in the cytoplasm.

Artificial circRNAs engineered to contain an array of MREs can act as an miR-21 sponge. This kind of circRNA sponge sequesters miR-21 from targeted genes in a manner similar to antagomiRs, suppressing gastric carcinoma cell proliferation *in vitro* and inhibiting esophageal carcinoma tumorigenesis *in vivo* (12, 69). Preliminary data suggest that artificial circRNAs are a new class of miRNA inhibitors that can provide novel future miRNA-based therapy in OS (Figure 1).

Natural circRNAs may be secreted and circulate in bodily fluids, and their circular structure gives them strong resistance to ribonucleases. Studies have partially proven that dysregulated circRNA expression can serve as a novel biomarker for OS. However, there are still some limitations before clinical application. First, potential circRNA candidates should be confirmed using larger sample sizes. Second, high specificity and high sensitivity must be emphasized. Third, the mechanism by which circRNAs are dysregulated in OS needs to be explored.

In conclusion, the dysregulation of some circRNAs plays a pathogenic role in OS. CircRNAs can function as tumor activators or suppressors in OS and have been further implicated in regulating OS chemoresistance and metastasis. Current treatments fail to further benefit patients with progressed OS due to chemoresistance, early metastasis, and a high recurrence rate. The use of RNAs as therapeutics has advanced tremendously and may provide a novel way to treat OS. For one thing, natural circRNAs can serve as tumor-suppressive molecules, such as hsa_circ_0002052, and could be natural anticancer agents when overexpressed in OS. For another, exogenous circRNAs can function as miRNA sponges to stop the cancer process, whether chemically synthesized or expressed by gene vectors. Hence, we are convinced that well-engineered circRNAs with multiple miR-21 binding sites may inhibit OS progression, since miRNA-21 is an essential onco-miRNA in OS. Although circRNA-based therapies for OS are still in the early stages, further development of circRNAs as biomarkers and miRNA inhibitors has a bright future.

## Author Contributions

DC, RW, and WL conceptualized the ideas. BW and HH contributed equally to this review. BW summarized related publications and drafted the manuscript. BW and HH created the figure and table. DC, RW, HH, and WL reviewed the manuscript and provided feedback. All authors have read and approved the final manuscript.

### Conflict of Interest

The authors declare that the research was conducted in the absence of any commercial or financial relationships that could be construed as a potential conflict of interest.
